# Biosurfactant Production by *Bacillus amyloliquefaciens* C11 and *Streptomyces lavendulae* C27 Isolated from a Biopurification System for Environmental Applications

**DOI:** 10.3390/microorganisms10101892

**Published:** 2022-09-23

**Authors:** M. Cristina Diez, Cesar Llafquen, Paola Fincheira, Claudio Lamilla, Gabriela Briceño, Heidi Schalchli

**Affiliations:** 1 Chemical Engineering Department, University of La Frontera, Av. Francisco Salazar 01145, Temuco 4780000, Chile; 2 Biotechnological Research Center Applied to the Environment (CIBAMA-BIOREN), University of La Frontera, Av. Francisco Salazar 01145, Temuco 4780000, Chile

**Keywords:** biosurfactants, *Bacillus*, lipopeptide, *Streptomyces*, glycolipids, environmental applications

## Abstract

Biosurfactant-producing bacteria can be found in contaminated environments such as biopurification systems (BPS) for pesticide treatments. A total of 18 isolates were screened to determine their ability to produce extracellular biosurfactants, using olive oil as the main carbon source. Out of the eighteen isolates, two strains (C11 and C27) were selected for biosurfactant production. The emulsification activities of the C11 and C27 strains using sunflower oil was 58.4 and 53.7%, respectively, and 46.6 and 48.0% using olive oil. Using molecular techniques and MALDI-TOF, the strains were identified as *Bacillus amyloliquefaciens* (C11) and *Streptomyces lavendulae* (C27). The submerged cultivation of the two selected strains was carried out in a 1 L stirred-tank bioreactor. The maximum biosurfactant production, indicated by the lowest surface tension measurement, was similar (46 and 45 mN/m) for both strains, independent of the fact that the biomass of the *B. amyloliquefaciens* C11 strain was 50% lower than the biomass of the *S. lavendulae* C27 strain. The partially purified biosurfactants produced by *B. amyloliquefaciens* C11 and *S. lavendulae* C27 were characterized as a lipopeptide and a glycolipid, respectively. These outcomes highlight the potential of the selected biosurfactant-producing microorganisms for improving pesticides’ bioavailability and therefore the degradational efficacy of BPS.

## 1. Introduction

Biosurfactants are surfactants that enable the properties of water and other fluids, such as the surface tension, to be changed [1]. Biosurfactants are primarily classified by their chemical structure and microbial origin. They are formed by a hydrophilic component that includes an acid, cations or anions, and mono, di, or polysaccharides. Meanwhile, the hydrophobic component is formed by chains of hydrocarbons or saturated or unsaturated fatty acids [2]. There are five major classes: glycolipids, lipopeptides, phospholipids, polymeric compounds, and neutral lipids [3]. Additionally, they can be grouped into high- and low-molecular-weight molecules. Biosurfactants with low molecular weights include glycolipids, lipopeptides, and phospholipids, while high-molecular-weight surfactants include particulate and polymeric surfactants [1]. Low-molecular-weight biosurfactants are used more often than high-molecular-weight biosurfactants due to their high surface-tension-reduction potential [4]. 

Biosurfactants of microbial origin could possibly replace synthetic surfactants because they have the properties of solubility, detergency, resistance to water hardness, and emulsifying, dispersing, and wetting capacity [5]. These biosurfactants can be applied in a wide variety of productive sectors, such as in the formulation of detergents, cosmetics, body hygiene products, paints and varnishes, agricultural applications, and pharmaceuticals, among others [6]. An example of an environmental application of biosurfactants is their use in the bioremediation of heavy metals [7], oil spills [8], and pesticides [9,10] due to their ability to solubilize hydrophobic compounds through the formation of emulsions that are formed by reducing the surface tension and interfacial tensions between liquids, solids, and gases [11]. In this process, molecular aggregates (micelles) are formed, which promote mass transfer to the microorganism. In the first instance, the "micelle" solubilized with the substrate (pesticide) is transported. Then, the exchange of the biosurfactant molecules with the cell occurs; this is where the degradation process occurs, i.e., the transfer of the substrate to the bacterial cell is finally carried out [12]. Many studies have examined pesticide biodegradation supported by biosurfactants from bacterial consortia such as surfactin [13] and rhamnolipid for chlorpyrifos (CHL) degradation [14], the solubilization of chlorinated hydrocarbon [15], and ethyl parathion, trifluralin, and methyl parathion [16]. 

Biosurfactants are produced from different microorganisms such as bacteria, yeast, and fungi [17,18,19]. Under certain conditions, many microorganisms can be induced to produce extracellular biosurfactants, with bacteria of the genera *Pseudomonas* and *Bacillus* being the best biosurfactant producers [20]. However, over the last few years, species of the genus *Streptomyces* have been used to produce biosurfactants [19,21,22]. 

The isolation and selection of different bacteria-producing biosurfactants and bioemulsifiers have been reported from a variety of environments, including a novel *Streptomyces* strain isolated from Antarctic soil [22], a *Streptomyces* sp. strain isolated from lichens in the Amazon region [19], *Pseudomonas* isolated from CHL-enriched soil [23], *Burkholderia* sp. isolated from oil-contaminated soil [24], *Bacillus amyloliquefaciens* and *Bacillus subtilis* isolated from contaminated soil [20], *Pseudomonas* and *Rhodococcus* isolated from a pesticide treatment system [25] and oil spills [8], *Bacillus* and *Pseudomonas* isolated from weathered oil-contaminated soil containing heavy metals [26], *B. subtilis* isolated from marine soil sediment [27], and halotolerant bacteria isolated from karst sinkholes [28], among others.

Biosurfactants synthesized from microbial cells vary greatly in their composition and thus their chemical and physical properties [29,30]. In this context, the production of biosurfactants is influenced by the effects of different operating factors such as the carbon/nitrogen ratio, pH, divalent cations, the specific substrate’s availability, and fermentation conditions, among others [31,32,33]. The production of biosurfactants in shake-flask experiments demonstrated that changing the carbon source employed affected both the amount of biomass produced and the biosurfactant secretion by *Pseudomonas aeruginosa* PG201 [29]. The authors found that using olive oil as the carbon source in a mineral salt medium provided the highest biosurfactant yield due to its high hydrophobic lipid content. Similar results were reported by Haba et al. [34], who used olive oil as the sole source of carbon for the growth of *Pseudomonas* sp. 

In general, ecological niches contaminated with hydrocarbon are the most commonly recommended sites for the isolation of biosurfactant-producing microorganisms for environmental remediation [35], and many studies have been carried out in this context. However, studies regarding the production of biosurfactants via pesticide-degrading microorganisms isolated from pesticide-treatment technologies such as biopurification systems (BPSs) are scarce, and the effectivity of these systems in which high concentrations of pesticides are treated needs to be improved. The use of pesticides has increased notably since the 1990s, increasing from 2.2 million tons in 1990 to 3 million in 2000 and exceeding 4 million tons in 2018 (FAOSTAT, 2020) [36]. The insecticide CHL, the herbicide atrazine (ATZ), and the fungicide iprodione (IPR) have been widely applied worldwide; they have relatively high chemical and biological stability in soils and aquifers and negative impacts on the environment, human health [37], and non-target organisms [38]. These pesticides are commonly used in Chile for the treatment of pests in farming [39], and their characteristics were reported by Levio et al. [40]. CHL has a low solubility in water (<2 mg L^−1^), whereas IPR and ATZ have solubilities of 6.8 and 35 mg L^−1^, respectively. The widespread use and toxicity of these pesticides means it is extremely necessary to increase the efficiency of BPSs by supplementing them with biosurfactants as an alternative to increase pesticides’ solubility and subsequent degradation. Therefore, the aim of this study was to isolate and characterize biosurfactant-producing bacteria from a BPS used for pesticide treatment at high concentrations. In addition, the production of biosurfactants by selected strains at the bench scale and the characterization of the biosurfactants obtained was carried out.

## 2. Materials and Methods

### 2.1. Bacteria Isolation and Selection 

Strains were isolated from a BPS that has been used during the last three years for the treatment of a mixture of the pesticides ATZ, CHL, and IPR at 50 mg kg^−1^ a.i. each, with re-application every 30 d [41]. To isolate the strains, samples were taken from different sections of the BPS (15–30 and 60 cm depth), mixed well, placed in sterile plastic bags, and stored at 4 °C for no longer than 12 h until their use. About 1 g of the biomixture sample was weighed and transferred to a Schott flask with 100 mL of sterile saline solution and placed on a rotary shaker at 120 rpm for 30 min. Serial dilutions were made up to 10^−5^ [42]. Then, 100 µL aliquots of the appropriate dilution were applied to the solid Luria Bertani (LB) medium and M1 agar. Plates were incubated at 28 ± 2 °C for 24 h. 

Bacterial colonies were preliminarily selected based on their colony morphology, Gram stain, and extracellular hydrolytic enzymes. For the enzymatic test, strains were inoculated in R2A agar (Difco Laboratories, Detroit, MI, USA) supplemented with starch (0.4% *w/v*), carboxymethylcelullose (0.4 *w/v*), tween 80 (1% *v/v*), skim milk (1% *w/v*), and gelatin (1% *w*/*v*) to determine the amylase, cellulase, lipase, protease, and gelatinase activity levels, respectively. After 72 h at 28 ± 2 °C, a positive reaction was observed through the visibility of transparent zones around the colonies or through the precipitation or coloration of the non-degraded substrate. For the detection of amylase, cellulase, and gelatinase activity levels, the plates were stained with Lugol’s solution, Congo red, and Frazier reagent, respectively. 

In addition, bacterial strains grown in LB medium were analyzed to determine their preliminary biosurfactant production by means of hemolytic activity in Trypticase Soy Agar (TSA) plates with 5% lamb blood (TSA; Fisher Scientific, Ottawa, ON, Canada) [22]. The plates were incubated for 72 h at 28 ± 2 °C. For the hemolytic test, the following results were considered: beta (β)—complete hemolysis; alpha (α)—incomplete hemolysis; gamma (γ)—no hemolysis. 

### 2.2. Selection of Biosurfactant-Producing Bacteria

Two strains based on the enzymatic and hemolytic activities were selected to evaluate their biosurfactant production using Bushnell–Haas (BH) broth (100 mL in 250 mL Erlenmeyer flasks) enriched with olive oil (2% *v*/*v*) as the carbon source. The flasks were inoculated (1% *v/v*) with fresh (48 h) bacterial culture and incubated at 28 ± 2 °C in a shaker (Labwit, Shanghai, China) at 150 rpm for 96 h. Biosurfactant production was verified in cell-free supernatant (CFS) (centrifugation at 10,000 rpm for 10 min at 4 °C) via drop-collapse and oil displacement. The positive control was commercial Tween 80 (2%) (Sigma-Aldrich, St. Louis, MO, USA), and the negative control was BH medium without a carbon source. The Emulsification activity (E24) of the biosurfactants produced was tested with two oils (olive and sunflower oil), and the stability of the biosurfactant was assessed in CFS from 24 h to 72 h by tracking the E24.

The E24 activity was determined as described by Cooper and Goldenberg [43]. A sample (2 mL) of CFS was mixed separately with olive oil and sunflower oil (2 mL each). The solution was mixed thoroughly for 2 min in a vortex mixer and left to stand for 24 h at room temperature. The E24 was expressed as the percentage of the emulsified layer height (mm) divided by the total of the liquid column height after 24 h and was calculated using the following equation:

E24 (%) = (HE)/Hs × 100%(1)

where HE is the height of the emulsion layer (mm) and Hs is the height of the total solution (mm).

### 2.3. Strains Identification

The selected strains were identified via molecular biology (16S rRNA) and matrix-assisted laser desorption/ionization time-of-flight (MALDI-TOF). The morphology was evaluated using optical and electronic microscopy (cells previously grown in LB medium) and biochemical characterization via the ApiZym® test. For electronic microscopy, a Scanning Electronic Microscope (SEM) (Model SU-3500 (Hitachi, Tokyo, Japan) was used. Fresh cells grown in LB medium (24 h of incubation at 28 ± 1 °C) were washed three times with distilled water and acetylcholine (0.1%). Then, the samples (65 µL) were placed in fluorodish plates, dried for 30 min at room temperature, and observed using SEM. 

#### 2.3.1. Bacterial Identification by Molecular Biology 

Genomic DNA was extracted using the UltraClean Microbial DNA Isolation Kit (MOBIO, Carlsbad, CA, USA). The 16S rRNA genes were selectively amplified from genomic DNA via polymerase chain reaction (PCR) using the universal primers 27F (5′-AGAGTTTGATCCTGGCTCAG-3′) and 1492R (5′-GGTTACCTTGTTACGACTT-3′) (IDT) [44,45]. PCR amplification was performed in a Multigene Optimal Thermal Cycler (Labnet, Edison, NI, USA). The PCR products were assessed via electrophoresis on 1% agarose gel stained with gel red. Sequencing was carried out by Macrogen (Seoul, Korea). The nearest taxonomic groups were identified with 16S rRNA nucleotide sequence BLASTN using DDBJ/EMBL/GenBank nucleotide sequence databases. The phylogenetic affiliation of bacteria in GenBank was determined using MEGA version X software [46].

#### 2.3.2. Bacterial Identification via MALDI-TOF/TOF MS

For MALDI-TOF/TOF MS analysis, 1 µL of the dry C11 and C27 strains was applied on the equipment plate (Bruker Daltonics, Bremen, Germany) and coated with 1 µL of a saturated solution of α-cyano 4-hydroxy cinnamic acid. Mass spectra were obtained with a smart beam laser source (334 nm). The analyses were performed with positive polarity, 20 kV acceleration voltage, and extraction with a 220 ns delay. Each spectrum was collected as an average of 1200 laser shots in the range of 2000 to 20,000 *m/z*. The equipment was calibrated with a protein calibration standard I (insulin, ubiquitin, cytochrome C, and myoglobin). The strain analyses were performed with the MALDI Biotyper Compass 4.1 software (Bruker Daltonics, Bremen, Germany) in the range of 3000–15,000 *m/z*. A score of ≥2 denoted identifications to the species level, and an intermediate log score between <2 and ≥1.7 denoted identification to the genus level.

### 2.4. Biosurfactant Production at Bench Scale 

The selected bacterial C11 and C27 strains were used for biosurfactant production in a bench-scale bioreactor. Biosurfactant production was carried out in a laboratory bioreactor with working volume of 1 L. Sterilized BH medium supplemented with olive oil (2% *v/v*) was inoculated with fresh (48 h) cultures of the selected strains (10% *v/v*) and incubated at room temperature for 96 h at a stirring speed of 200 rpm. Biosurfactant production was evaluated in CFS by means of oil displacement, superficial tension, and E24. 

### 2.5. Biosurfactant Extract Preparation and Characterization 

Biosurfactant characterization was achieved in the crude biosurfactant extract (CBE), which was obtained as described by Lourenço et al. [47] with some modifications. Briefly, the extraction was carried out by mixing previously acidified (6N HCl to pH 2.0, stored overnight at 4 °C) CFS (obtained from the bioreactor) with a mixture of chloroform–methanol 2:1 (*v/v*). The mixture was stirred at 200 rpm at room temperature (25 °C) for 3 h. Then, it was centrifuged, and the supernatant was evaporated in a rotary vacuum evaporator and stored at −18 °C. The chemical characterization of the CBE was performed via thin-layer chromatography (TLC) and Fourier Transform Infrared Spectroscopy (FTIR) [18].

### 2.6. Analyses

#### 2.6.1. Drop-Collapse

Drop-collapse was achieved as described by Jain et al. [48]. A 25 µL aliquot of CFS from each sample was pipetted onto a drop of distilled water (25 µL) placed onto a Parafilm M® (Heathrow Scientific, Vernon Hills, IL, USA) sheet. The drop flattened and spread over the next 60 s. Tween 80 (Sigma-Aldrich) and distilled water were used as the positive and negative controls, respectively. 

#### 2.6.2. Oil Displacement Test

The oil displacement assay was performed as described by Morikawa et al. [49] with some modifications. Briefly, each oil aliquot (25 µL) was placed on the surface of distilled water (40 mL) in a Petri dish (110 mm). Then, the clear zone of oil displacement was visualized after the addition of 25 µL of CFS to the center of the oil film. The results were expressed as the diameter (mm) of the halo displacement obtained after 3 s. Distilled water was employed as the negative control, and Tween 80 was used as the positive control. 

#### 2.6.3. Superficial Tension

The superficial tension was measured in 20 mL of the CFS of the culture medium after 96 h of incubation using a tensiometer (HZZL-3 Transformer Oil Tension Tester, Huazheng, Baoding, China) at room temperature. The validity of this analysis was confirmed with distilled water (72 mN/m) before each reading.

#### 2.6.4. Biosurfactant Crude Extract Characterization 

The qualitative analysis of the biosurfactant’s chemical composition was conducted using TLC plates (Silica gel 60 F254; Merck, Darmstadt, Germany). Drops of BCE dissolved in methanol were placed on TLC plates with chloroform:methanol:acetic acid (65:15:2 *v/v*) as the mobile phase. Plates were exposed to iodine vapors for lipid detection, sprayed with 50% (*v*/*v*) H_2_SO_4_ for carbohydrate detection, and exposed to ninhydrin solution 2% for amino acid visualization [18]. During FTIR spectroscopy, about 2 mg of freeze-dried extract was milled with 200 mg of KBr and compressed into a thin pellet. The FTIR spectra were obtained by measuring the transmittance between the frequencies of 4000 to 400 cm^−1^ with a resolution of 2 cm^−1^ [38]. The biosurfactant concentration expressed in terms of critical micellar concentration (CMC) was estimated by measuring the surface tension in a biosurfactant (Huazheng) with the biosurfactant crude extract with varying dilutions using the Du Noüy ring method [50]. 

### 2.7. Statistical Analysis

All the experiments were carried out in triplicate. The results regarding the growth behavior and biosurfactant production of the selected strains were analyzed by multiple comparisons of means, performing a one-way analysis of variance (ANOVA) and a Tukey test (*p* < 0.05), and using GraphPad Prism 8.0 (Graphpad Software, San Diego, CA, USA). 

## 3. Results

### 3.1. Bacteria Isolation and Selection

A total of 18 bacterial strains were isolated from a BPS biomixture. Based on the morphological and phenotypic characterization, we found fourteen rod-shaped Gram (+) strains (77.7%), three Gram (−) strains (16.7%), and one round-shaped bacterial strain (5.6%) (Table 1). Some of them presented red (C23), yellow (C26), light pink (C14, C15, C16, and C25), and dark pink (C27) pigmentation. 

The extracellular hydrolytic activity levels of the isolated bacterial strains are shown in Table 1. Gelatinase and protease activity levels larger than or equal to 1.1 were found in seven and six strains, respectively. The enzymatic activity levels of other components were found to be poor in the tested strains. Furthermore, the hemolytic activity was analyzed to determine the level of preliminary biosurfactant production in the TSA plates with 5% Lamb blood. Two strains (C11 and C27) showed high levels of hemolytic activity (β), the other two strains (C16 and C25) presented low levels of hemolytic activity (α), and the remaining strains showed negative hemolytic activity (γ) (Table 1). Therefore, based on the enzymatic and hemolytic activity levels, strains C11 and C27 were selected to evaluate their levels of biosurfactant production.

### 3.2. Biosurfactant Production

The selected strains, C11 and C27, were tested to determine their biosurfactant production by using a BH medium with olive oil. The biosurfactant production was verified in CFS via drop-collapse and oil displacement. Both of the strains showed a positive reaction (+) in the drop-collapse test. However, the C27 strain showed the highest level of oil displacement (11 cm) compared with the C11 strain (5.5 cm) when grown with olive oil as the carbon source.

**Figure 1:**
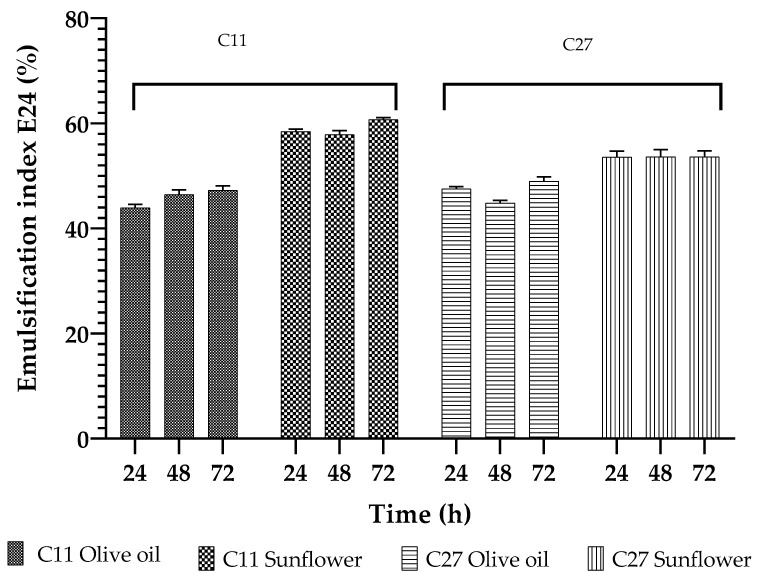
Emulsification index (E24) (%) of the biosurfactants produced with *Bacillus amyloliquefaciens* C11 and *Streptomyces lavendulae* C27 strains stored for 24, 48, and 72 h and tested with olive and sunflower oil. There were no significant differences between treatments (*p* < 0.05).

The E24 index was determined to verify the biosurfactant activity of the selected strains in two oils (olive and sunflower oil). The results showed that the E24 values of the biosurfactants produced by both strains were highest when sunflower oil was used in the test (58.4 and 53.7% for the C11 and C27 strains, respectively) (Figure 1). The E24 values were always lower when olive oil was used in the test rather than sunflower oil; a maximum of 46.6 and 48.0% was obtained for the C11 and C27 strains, respectively. With respect to the stability, the biosurfactants produced by both strains exhibited a high level of stability during storage, showing no significant differences (*p* < 0.05) when stored for up to 72 h (Figure 1).

### 3.3. Strains Identification and Characterization

**Figure 2:**
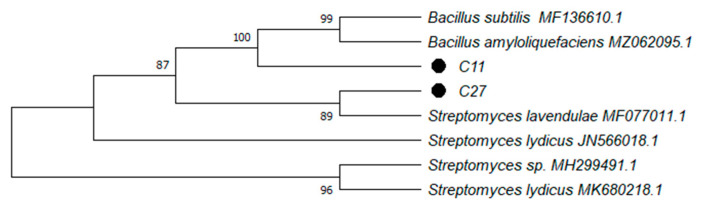
Neighbor-joining dendrogram based on 16S rRNA sequence of *Bacillus amyloliquefaciens* C11 strain and *Streptomyces* *lavendulae* C27 strain.

The molecular identification of the C11 and C27 strains through 16S rRNA gene sequencing was carried out (Table 2). The C11 strain had a clear phylogenetic relationship with the phylum *Firmicutes* genus *Bacillus* of 96.7% and the closest species relationship with *B. amyloliquefaciens*, showing a 100% identity according to the Basic Local Alignment Search Tool (BLAST). The C27 strain showed a 100% identification with the species *Streptomyces lavendulae*; therefore, it had a close phylogenetic relationship with the phylum *Actinobacteria* and the genus *Streptomyces* (Figure 2). The analyses conducted via MALDI-TOF also showed high scores for *Bacillus* sp. and *Streptomyces* sp. (Table 2).

**Figure 3:**
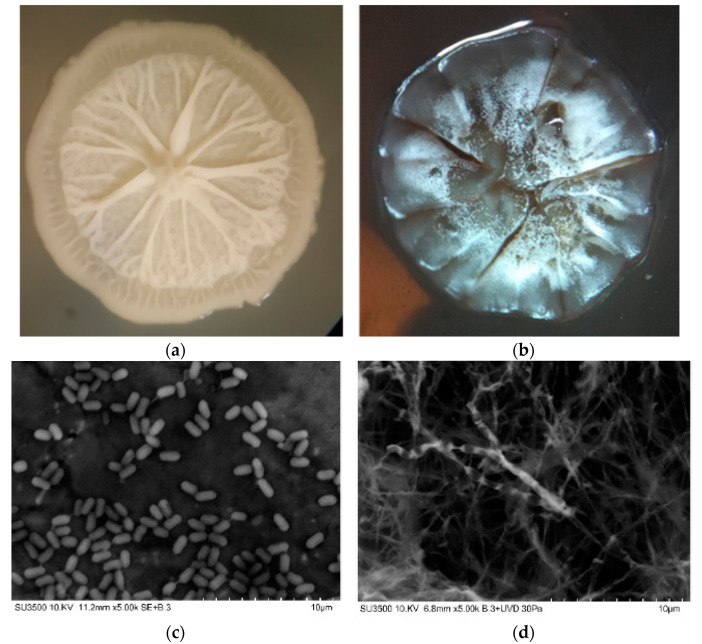
Optical and SEM images of *Bacillus amyloliquefaciens* (**a**,**c**) and *Streptomyces lavendulae* (**b**,**d**).

The morphological characteristics of the selected bacterial strains confirmed the results of the molecular identification via 16sRNAr and MALDI-TOF, as shown in Figure 3. The *B. amyloliquefaciens* C11 strain was shown to have a rod-shaped morphology when visualized via SEM, while the *S. lavendulae* C27 strain had typical aerial branched hyphae.

Extracellular enzymes of the *B. amyloliquefaciens* C11 and *S. lavendulae* C27 strains were evaluated using the ApiZym® test (Table 3). Enzymatic potential was detected within the tested strains. It was found that the *B. amyloliquefaciens* C11 strain showed high levels of alkaline phosphatase, esterase (C4), esterase lipase (C8), lipase (C14) leucinearylamidase, valinearylamidase, acid phosphatase, naphthol-AS-BI-phosphohydrolase, and N-acetyl-glucoseamidase activity. On the other hand, the *S. lavendulae* C27 strain showed high levels of alkaline phosphatase, esterase leucinearylamidase, valinearylamidase, trypsin, α-quimotrypsyne, acid phosphatase, naftol-AS-BI-phosphohydrolase, and glucosidase (α and β) activity.

### 3.4. Biosurfactant Production at Bench Scale

**Figure 4:**
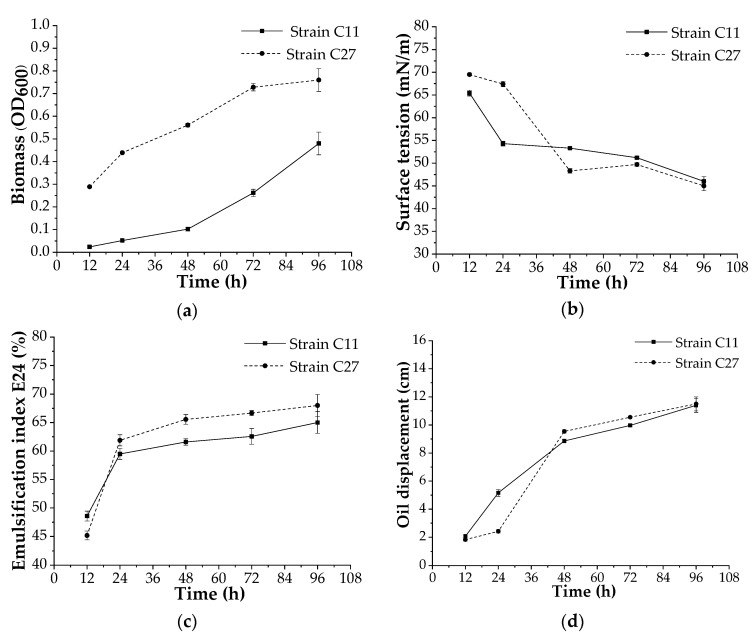
Growth behavior and biosurfactant production via the selected strains *B. amyloliquefaciens* C11 and *S. lavendulae* C27 in a bench-scale bioreactor. (**a**) biomass; (**b**) surface tension; (**c**) emulsification index E24; (**d**) oil displacement.

The bacterial strains *B. amyloliquefaciens* C11 and *S. lavendulae* C27 were tested to determine their biosurfactant production in a bench-scale bioreactor. The growth behaviors of both strains in the bioreactor were different. The *B. amyloliquefaciens* C11 strain grew more slowly and showed a more pronounced phase lag compared to the *S. lavendulae* C27 strain (Figure 4a).

The production of biosurfactants using the two strains was demonstrated from the beginning of growth until the end of the assay, reaching similar values for surface tension of 46 and 45 mN/m for the *B. amyloliquefaciens* C11 and *S. lavendulae* C27 strains (Figure 4b). In addition, E24 values of 65% and 68% were observed in the biosurfactants produced using the *B. amyloliquefaciens* C11 and *S. lavendulae* C27 strains, respectively, after 92 h of incubation (Figure 4c). On the other hand, the biosurfactant activity, which was measured using the oil displacement in both strains, increased as the incubation time increased; similarly, high values (up to 11.5 cm) were obtained after 96 h of incubation (Figure 4d). 

### 3.5. Biosurfactant Characterization

The chemical compositions of the biosurfactants evaluated in the CBE of the selected strains, *B. amyloliquefaciens* C11 and *S. lavendulae* C27, were obtained via TLC. The presence of peptides and lipids was verified in the *B. amyloliquefaciens* C11 strain (Figure S1), suggesting the presence of a lipopeptide-type biosurfactant [51]. On the other hand, the presence of lipids and carbohydrates was observed in the *S. lavendulae* C27 strain (Figure S1), suggesting the presence of a glycolipid-type biosurfactant [52]. 

**Figure 5:**
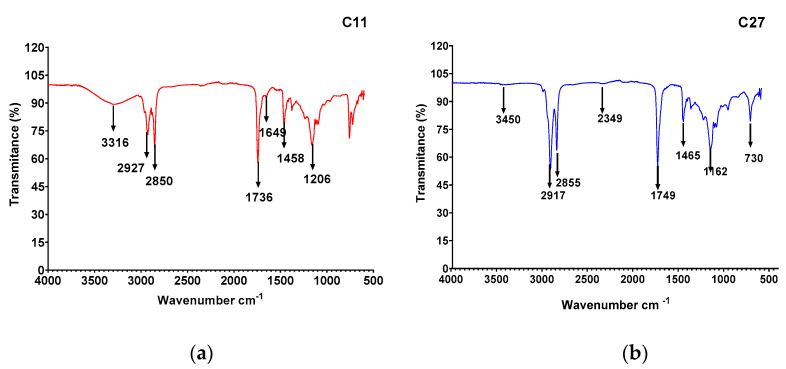
FTIR spectra of biosurfactants produced using (**a**) *B. amyloliquefaciens* and (**b**) *S. lavendulae* grown in Bushnell–Haas medium supplemented with olive oil.

The FTIR analyses of the CBE for the *B. amyloliquefaciens* C11 and *S. lavendulae* C27 strains are shown in Figure 5a,b. The CBE spectra for *B. amyloliquefaciens* C11 showed the characteristic bands of peptide component molecules at 3300–3400 cm^−1^ (N-H stretching mode), 1650–1700 cm^−1^ (CO–N bond stretching mode), and at 1520–1550 cm^−1^, attributable to the N–H bond stretch mode combined with the C–N stretch mode. On the other hand, the presence of an aliphatic chain indicating C–H modes at 2840–3000 cm^−1^ was also observed. The absorbance at 1620–1660 cm^−1^ belonged to the C=O stretching vibration of the amide I region, while a band observed at 1735–1750 cm^−1^ was due to a carbonyl group. Therefore, the biosurfactant produced by the C11 strain was expected to contain fatty acids and peptide moieties, revealing the lipopeptide nature of the biosurfactant. The analysis of the CBE via infrared spectroscopy for the *S. lavendulae* C27 strain showed that the most important peak was located at 2917 cm^−1^. Bending vibrations of the methyl group were shown at 1749 cm^−1^. Stretching of the C=O double bond of esters and carboxyl groups was shown at 1566 cm^−1^, and C-H (fatty acids) peaks were revealed at 730 cm^−1^, which demonstrated CH_2_CH_3_ stretching and CO stretching.

## 4. Discussion

To obtain specialized biosurfactant-producing microorganisms, different environments have been studied, which have mainly been associated with oil-contaminated environments. In the presence of contaminants such as pesticides, some microorganisms can be induced to produce extracellular biosurfactants. In previous studies, Briceño et al. [53] identified pesticide-tolerant and pesticide-degrading strains from a BPS, and some of them were later identified as biosurfactant producers [25]. In our study, we screened novel bacterial strains isolated from a BPS biomixture to determine their biosurfactant production and possible subsequent cultivation in a bench-scale bioreactor.

Of the eighteen bacterial strains tested, we selected two bacteria according to their enzymatic profile and high (β) level of hemolytic activity. The response obtained from the C11 and C27 strains with qualitative hemolytic activity is a good preliminary indicator of biosurfactant production by microorganisms such as that described for both fungi and bacteria [54]. The molecular, morphological, and biochemical characteristics indicated that the selected strains belonged to the genera *Bacillus* and *Streptomyces*, which have been previously reported by other authors as biosurfactant-producing bacteria [19,55]. Some of these include *B. subtilis* and *B. safensis* J2, which have the potential to be applied in oil recovery and the restoration of diesel-contaminated soil [56,57]; Actinobacteria such as *S. griseoplanus* NRRL-ISP5009, which was isolated from oil-contaminated soil and was able to produce a biosurfactant with emulsification activity [58]; and *Streptomyces* sp. R1, which was able to produce a biosurfactant with potential industrial applications [21]. The morphological characterization of the colony of *B. amyloliquefaciens* C11, cultivated in the LB culture medium, showed an irregular margin and a “jellyfish head”, characteristic of strains belonging to the *Bacillus* genus. A similar colony morphology for this type of *Bacillus* was reported by Liu et al. [59]. For *S. lavendulae* C27, a dark-gray to black pigmentation was noted within 5 d of growth. In an M1 medium, a white mycelium was observed with changes in its pigmentation over time but drops of exudate were present on the surface of the colony. The bacteria *S. lavendulae* has been reported to be present in soils worldwide, and it is known for its production of biologically active metabolites with medical and biotechnological applications [60]. In addition, the morphology visualized via SEM showed that *B. amyloliquefaciens* C11 also has a typical *Bacillus* rod-shaped morphology (0.9 μm width and 1.4 μm length, in average), which is similar to the results reported by Kadaikunnan et al. [61] for *B. amyloliquefaciens* VJ-1. The SEM analysis of *S. lavendulae* C27 showed typical *Streptomyces* aerial branched hyphae. The enzymatic characterization of the *B. amyloliquefaciens* C11 strain showed similar positive reactions to amylase, phosphatase, lipase, and protease enzymes, similar to the results obtained by Kakou et al. [62], who evaluated seven species of *Bacillus*, including *B. amyloliquefaciens*. Overall, *Bacillus* species have been shown to have good α-glucosidase (100%) and ß-glucosidase (94%) synthesis abilities. Contrarily, our *B. amyloliquefaciens* C11 strain did not produce **α**-glucosidase and β-glucosidase, which may have been due to the fact that olive oil was used as a source of carbon, and not a carbohydrate. Finally, the characterization conducted through an ApiZym® test showed the presence of several enzymes in *S. lavendulae* C27, which are associated with various functions and biotechnological applications [63]. 

The biosurfactant production of the strains was evaluated preliminary by means of oil displacement and E24 in the culture medium with olive oil (2% *v/v*) as the carbon source. The free fatty acid content of olive oil supports the growth of microorganisms, stimulating biosurfactant production [32]. An oil displacement test is an indirect measurement of a surfactant’s surface activity on oil; a larger clear zone diameter indicates the higher surface activity of the tested solution [10]. In our study, the *B. amyloliquefaciens* C11 and *S. lavendulae* C27 strains demonstrated oil displacements with diameters >5.5 cm as well as high E24 values >45% for olive oil and >53% for sunflower oil, which were similar values to those found in the literature [64]. According to the literature, the emulsification index varies depending on the type of oil used for the analysis [19,25,32]. Santos et al. [19] determined the E24 values of a biosurfactant from *Streptomyces* sp. DPUA1559 of 47, 41, 36, and 30% for corn oil, soybean oil, canola oil, and sunflower oil, respectively. In addition, Abouseoud et al. [32] found that diesel oil and kerosene were the best substrates (55%) and sunflower oil was a less successful substrate for emulsification (45%) with a biosurfactant produced using *Pseudomonas fluorescens*.

The study conducted in a bench-scale bioreactor confirmed our previous results, and both strains produced surfactants independent of the biomass amount. In accordance with our previous observations, the surfactant showed a similar response, which was tested by means of oil displacement and E24. In addition, the surfactant was as acceptable in terms of its stability, because the emulsion activity was maintained over time, and this has also been observed in other surface-active compounds produced using actinobacteria [60]. With regard to the surface tension, the value decreased by up to 46 and 45 mN/m with the *B. amyloliquefaciens* C11 and *S. lavendulae* C27 strains, respectively. The values, although high, are in the range of the values of biosurfactants produced by other microorganisms under non-optimized conditions [8,10,13,21]. The ability to lower the surface tension of aqueous solutions is an important property that characterizes a potent surface-active agent [65], and according to the literature, a drop in surface tension to under 35 mN/m indicates that the microorganism is an efficient biosurfactant producer [54]. A reduction in surface tension to 22.9 mN/m with surfactants produced using *B. amyloliquefaciens* SAS-1 [56] and 27 mN/m with the biosurfactant produced using *Bacillus* sp. [66] was reported. On the other hand, *Streptomyces* sp. DPUA1559, which was isolated from lichens in the Amazon region, showed surface tension reduction values of around 27.1 mN/m [19]. The low level of reduction in surface tension obtained with our isolates could have been due to the fact that we did not optimize the culture medium. Process parameters such as the pH of the medium; the temperature of incubation; aeration levels; the nutrient composition including nitrogen, carbon, and mineral sources; and salinity are very important factors for the optimization of the production of biosurfactants [27]. Therefore, our next step will be to optimize the surfactant produced using the selected microorganisms. 

The biosurfactant extracted from CFB was submitted to TLC and FT-IR. Lipopeptides are the most widely studied and characterized biosurfactants in numerous Bacillus species, including *B. subtilis* and *B. amyloliquefaciens* [55,59,66]. The biosurfactant produced using *B. amyloliquefaciens* C11 was expected to contain fatty acids and peptide moieties, revealing the lipopeptide nature of the biosurfactant (TLC analysis). The FTIR spectrum showed that it had similarities with lipopeptide biosurfactants and surfactin generally produced using *Bacillus* sp. [27,55,67]. On the other hand, the analysis of the CBE via TLC showed the glycolipid nature of the biosurfactant produced using the *S. lavendulae* C27 strain. When infrared spectroscopy was applied to the *S. lavendulae* C27 strain, the evidence of CH_2_CH_3_ stretching and CO stretching was revealed, probably corresponding to a fatty acid similar to a glycolipid produced by *Streptomyces luridus*, a strain of Antarctic origin [18]. The biosurfactant produced using *Streptomyces* sp. DPUA1559, which was isolated from lichens in the Amazon region, showed a single protein band, an acidic characteristic, and a molecular weight of around 14.3 kDa, suggesting its glycoproteic nature [15]. Most studies have effectively reported that biosurfactants produced using the genus *Streptomyces* sp. are glycolipids [19,52]. However, a surfactant based on lipopeptides was reported by Zambry et al. [21]. Santos et al. [65] reported an isolated biosurfactant produced using *Streptomyces* sp. DPUA1566 with a composition of 84% proteins and 15% lipids. 

## 5. Conclusions

Two biosurfactant-producing bacteria were selected from a biopurification system (BPS) used for pesticide treatment at high concentrations and identified as *Bacillus amyloliquefaciens* C11 and *Streptomyces* *lavendulae* C27. Biosurfactants produced with olive oil (2%) as the sole carbon source were characterized as lipopeptides from the *B. amyloliquefaciens* C11 and glycolipids from the *S. lavendulae* C27 via TLC and FTIR analysis. The biosurfactants produced using both strains showed high emulsification capacity (>65%) and adequate surface tension activity (46 and 45 mN/m), despite the fact that the cultivation conditions were not optimized. These results highlight the potential of the selected biosurfactant-producing microorganisms isolated from a BPS for improving pesticides’ bioavailability, and thereby the degradational efficacy of this system.

## Figures and Tables

**Table 1:** Morphological and enzymatic characteristics of the isolated bacteria.

Strain	Gram (+/−)	Shape of Cell	Enzymatic Activity (cm)					
			Gelatinase	Protease	Amylase	Lipolytic	Cellulase	Hemolytic
C11	+	Rods	2.2	2	<1	1.1	<1	++++ β
C12	+	Rods	1.1	-	-	-	-	γ
C13	+	Rods	1.3	1.5	-	<1	<1	γ
C14	+	Rods	<1	-	-	<1	<1	γ
C15	+	Rods	-	-	-	-	-	γ
C16	+	Rods	1.1	1.8	-	1	-	+ α
C17-1	−	Cocci	1.1	<1	-	-	<1	γ
C17-2	+	Rods	<1	-	-	<1	<1	γ
C18	+	Rods	1.1	1.6	-	<1	<1	γ
C19	+	Rods	<1	-	-	-	-	γ
C20	−	Rods	-	-	-	-	-	γ
C21	+	Rods	-	-	-	-	-	γ
C22	+	Rods	-	-	-	-	-	γ
C23	+	Rods	<1	<1	-	1.2	<1	γ
C24	+	Rods	1.1	1.5	-	-	<1	γ
C25	−	Rods	-	-	-	-	-	+ α
C26	−	Rods	<1	1.6	-	<1	1.7	γ
C27	+	Rods	<1	-	-	-	-	+++ β

Beta (β) = complete hemolysis; alpha (α) = incomplete hemolysis; gamma (γ) = no hemolysis. Gram (+/-) = Gram (positive/negative). Enzymatic activity - = not detected. + = smaller than 1 cm; +++ = between 1 and 4 cm; ++++ = larger than 4 cm.

**Table 2:** Strains’ identification by 16sRNAr and MALDI-TOF.

Strain	Strain Identity	Accession Number (Ribotyping)	MALDI-TOF Score
C11	*Bacillus amyloliquefaciens*	ON732850	2.520
C27	*Streptomyces lavendulae*	ON732851	2.000

**Table 3:** Biochemical characteristics of *B. amyloliquefaciens* C11 and *S. lavendulae* C27 strains.

Enzyme	*B. amyloliquefaciens* C11	*S. lavendulae* C27
Alkaline phosphatase	+	+
Esterase (C 4)	+	+
Esterase lipase (C 8)	+	-
Lipase (C 14)	+	-
Leucine arylamidase	+	+
Valine arylamidase	-	+
Cystine arylamidase	-	-
Trypsin	-	+
α-quimotrypsyne	-	+
Acid phosphatase	+	+
Naftol-AS-BI-phosphohydrolase	+	+
α-galactosidase	-	-
β-galactosidase	-	-
β-glucuronidase	-	-
α-glucosidase	-	+
β-glucosidase	-	+
*N*-acétil-β-glucosaminidase	+	-
α-mannosidase	-	-
α-fucosidase	-	-

+ = reaction positive; - = no activity.

## Data Availability

Not applicable.

## Supplementary Materials

The following supporting information can be downloaded at: https://www.mdpi.com/article/10.3390/microorganisms10101892/s1, Figure S1: TLC plates of purified biosurfactants of *Bacillus amyloliquefaciens* (a) and *Streptomyces lavendulae* (b) strains. Reaction with ninhydrin in (A), iodine in (B) and H_2_SO_4_ in (C).
