# Supplementary material for: Biosurfactant Production by Bacillus amyloliquefaciens C11 and Streptomyces lavendulae C27 Isolated from a Biopurification System for Environmental Applications
Source: Microorganisms. 2022 Sep 23;10(10):1892. doi: 10.3390/microorganisms10101892 (PMC9609857; doi:10.3390/microorganisms10101892)
Supplement: Supplementary file 1 [file microorganisms-10-01892-s001.zip › microorganisms-1864343-supplementary.pdf]

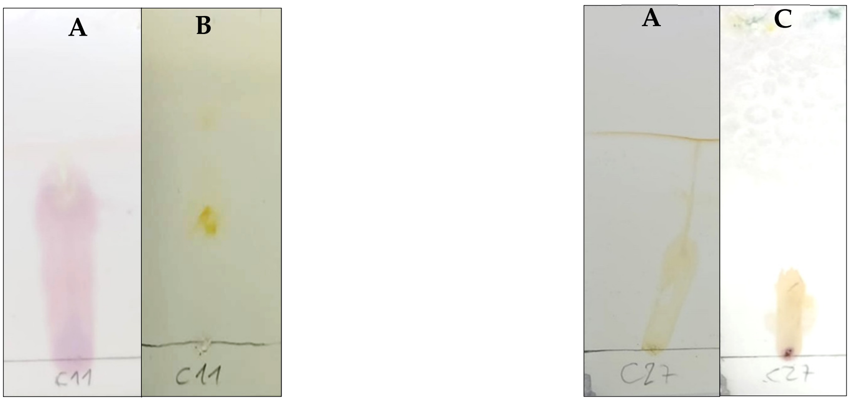

(**a**) (**b**)

Figure S1. TLC plates of purified biosurfactants of *Bacillus amyloliquefaciens* (**a**) and *Streptomyves. lavendulae* (**b**) strains. Reaction with ninhydrin in (A), iodine in (B) and H_2SO_4 in (C).
